# Clinical and biological characteristics and prognostic impact of somatic *GATA2* mutations in myeloid malignancies: a single institution experience

**DOI:** 10.1038/s41408-021-00517-0

**Published:** 2021-06-30

**Authors:** Ahmad Nanaa, David Viswanatha, Zhuoer Xie, Dragan Jevremovic, Phuong Nguyen, Mohamad E. Salama, Patricia Greipp, Kurt Bessonen, Naseema Gangat, Mrinal Patnaik, Animesh Pardanani, Hassan B. Alkhateeb, Mithun Shah, William Hogan, Ayalew Tefferi, Mark Litzow, Rong He, Aref Al-Kali

**Affiliations:** 1 Division of Hematology, Mayo Clinic, 200 First Street SW, Rochester, MN 55905 USA; 2 Division of Hematopathology, Mayo Clinic, 200 First Street SW, Rochester, MN 55905 USA; 3 Division of Cytogenetics, Mayo Clinic, 200 First Street SW, Rochester, MN 55905 USA

**Keywords:** Cancer genomics, Myelodysplastic syndrome

**Dear Editor,**

The *GATA2* gene, located on chromosome 3q21, is a member of the *GATA* family of six *GATA* transcription factors regulating gene expression via two conserved zinc finger (ZF) domains named after their corresponding location, N and C terminal ZF domains [1, 2]. *GATA2* regulates hematopoietic stem cell proliferation and differentiation [3, 4]. It regulates mononuclear cell development, alveolar macrophage activity, and plays a role in lymphatic development [5, 6]. Different location of *GATA2* mutation is associated with distinct phenotype and leukemogenic mechanisms, with ZF-2 domain mutations common in Monocytopenia and mycobacterial infection (MonoMAC) syndrome /Dendritic Cell, Monocyte, B and NK Lymphoid deficiency syndrome (DCML), familial myelodysplastic syndrome/acute myeloid, and chronic myeloid leukemia (CML) blast transformation [7, 8]. ZF1 mutations have been described in close association with biallelic *CEBPA* (bi*CEBPA*) mutations and French-American-British (FAB) M1 subtype in adult acute myeloid leukemia (AML) [9]. Nonsense or frameshift *GATA2* mutations reduce the overall expression of *GATA2* protein. This is in contrast to missense mutations affecting the *GATA2* ZF1 and ZF2 domains, which cause loss of function by impairing binding to *GATA*-DNA motifs [5]. Somatic *GATA2* mutation is found in 1–4% of patients with sporadic myeloid malignancies [10–12]. Greif et al. described *GATA2* mutation in 40.6% of 32 cytogenetically normal biCEBPA-mutated AML patients. All of the *GATA2* mutations were missense and located in the ZF-1 domain [13, 14]. Another study reported that most of *GATA2* mutations (66.7%) in biCEBPA AML occurred in the ZF1 domain [15].

We hypothesize that the *GATA2* mutation location and mutation type have distinct impact on clinical and biological features of myeloid neoplasms (MN), along with their interaction with other co-occurring somatic mutations. We retrospectively analyzed (after approval by our institutional review board) 3872 consecutive patients who had next-generation sequencing (NGS) testing between 5/2015 and 7/2020 at Mayo Clinic, and identified fifty-four MN patients harboring 63 *GATA2* mutations (Supplementary materials and methods). Unlike most somatic *GATA2* studies (limited to AML), our study included four different diagnostic groups; 15 (27.7%) with myelodysplastic syndromes (MDS), 16 (29.6%) with myelodysplastic/myeloproliferative neoplasms (MDS/MPN), 9 (16.6%) with myeloproliferative neoplasms (MPN), and 14 (25.9%) AML (Table 1). The median age was 67 years (range, 26–89) and 36 (67%) patients were males. The median white blood cell (WBC) was 12.9 × 10^9^/L, median hemoglobin (Hg) of 8.6 g/dl and platelets of 53 × 10^9^/L, respectively (Table 1 and Supplemental Table S1).

**Table 1:** Patients characteristics with GATA2 mutation.

**a**. Characteristics and hematological features of patients with *GATA2* mutation.					
Variable			Value		
No. of patients			54		
Age years, median(range)			67 (26–89)		
Sex (male), *n* (%)			36 (67)		
Hemoglobin G/DL, median (range)			8.6 (5.8–13)		
Leukocytes 10^9^/L, median (range)			12.9 (0.3–186)		
Thrombocytes 10^9^/L, median (range)			53 (1.5–873)		
MCV median (range)			93 (77–119)		
ANC, median (range)			6.64 (0.17–96.7)		
AMC, median (range)			0.39 (0–44.5)		
ALC, median (range)			1.44 (0.37–38.8)		
RDW, median (range)			17.7 (13.1–26.8)		
Number of mutations, median (range)			4 (1–6)		
Diagnosis					
MDS, *n* (%)			15 (27.7)		
MPN, *n* (%)			9 (16.6)		
AML, *n* (%)			14 (25.9)		
MDS/MPN, *n* (%)			16 (29.6)		
Mutation types					
Missense, *n* (%)			18 (33.3)		
In-frame deletion, *n* (%)			18 (33.3)		
Frameshift, *n* (%)			12 (22.2)		
Non-sense, *n* (%)			4 (7.4)		
Duplication that span through splicing site, *n* (%)			2 (3.7)		
Mutation location					
ZF1, *n* (%)			9 (17)		
ZF2, *n* (%)			31 (57)		
Non-ZF, *n* (%)			14 (26)		
Abnormal cytogenetics					
Yes, *n* (%)			32 (63)		
No, *n* (%)			19 (37)		
**b**. Summary of molecular findings according to *GATA2* mutation location.					
Location	*ASXL1* co-mutations (%)	Mutation type (%)		Unique co-mutations	
ZF-1	11	Missense (78%), duplication that span through splicing site (22%)		* CEBPA, KIT, SF3B1, TET2*	
ZF-2	74	In-frame deletion (58%), missense (35.5%), frameshift (6.5%)		* JAK2*	
Non-ZF	50	Frameshift (71%), non-sense (29%)		None	
**c**. Summary of molecular findings according to the diagnostic groups.					
Diagnosis	*GATA2* mutation location (%)	Most frequent *GATA2* mutation type (%)	Frequency of *ASXL1* co-mutations (%)	Unique co-mutations	Patients with several *GATA2* mutations (N)
MDS	ZF-1 (13%), ZF-2 (47%), Non-ZF (40)	Frameshift (33%), missense (33%)	47	* BCOR U2AF1*	1
MPN	ZF-1 (0%), ZF-2 (89%), Non-ZF (11%)	In-frame deletion (67%)	78	* CALR MPL JAK2*	2
AML	ZF-1 (43%), ZF-2 (36%), Non-ZF (21%)	Missense (50%)	14	* CEBPA KIT FLT3*	1
MDS/MPN	ZF-1 (6%), ZF-2 (69%), Non-ZF (25%)	In-frame deletion (38%)	94	* ASXL1 SETBP1 SRSF2*	4

*MCV* mean corpuscular volume, *ANC* absolute neutrophil count, *AMC* absolute monocyte count, *ALC* absolute lymphocyte count, *RDW* red cell distribution width, *MDS* myelodysplasia neoplasm, *AML* acute myeloid leukemia, *MPN* Myeloproliferative neoplasms, *MDS/MPN* Myelodysplastic/Myeloproliferative neoplasms, *ZF* zing finger.

The median variant allele frequency (VAF) of *GATA2* mutations was 31% (range, 5–74%), with a median VAF of 28, 37, 34, and 39% in MDS, MDS/MPN, MPN, and AML, respectively. There was no statistically significant difference between median VAF in the different diagnostic groups (*p* = 0.6). Seven patients harbored two, and one patient had three *GATA2* mutations. The most frequent variants were p.Lys390del (*N* = 5, 9%), p.Met388_Lys389del (*N* = 4, 8%) and p.Arg396Trp (*N* = 3, 6%), while other variants were detected in 1–2 patients (Fig. 1A and Supplemental Table S2). Thirty-one (57%) mutations clustered in ZF2, 9 (17%) in the ZF1 domain, and 14 (26%) were outside the ZF domains (non-ZF). ZF1 and non-ZF mutated patients had lower mean VAF compared to ZF2 patients (22% vs. 38%, *p* = 0.03) and (26% vs. 38%, *p* = 0.02), respectively. The most common *GATA2* mutation types were missense mutation (*N* = 18, 33.3%) and in-frame deletion (*N* = 18, 33.3%). Other less common mutation types were frameshift (*N* = 12, 22.2%), nonsense (*N* = 4, 7.4%), and duplication that span through splicing site (*N* = 2, 3.7%) (Supplemental Table S2). Duplication that spans through splicing site, in-frame deletion, and nonsense mutations occurred only in ZF1, ZF2, and non-ZF, respectively. ZF1 patients had significantly higher frequency of missense mutations compared to ZF2 patients (78% vs. 35%, *p* = 0.02). Frameshift mutation was more common in non-ZF patients compared to ZF2 patients (83% vs. 17%, *p* < 0.0001). AML patients had significantly more ZF1 mutations compared to chronic MN (43% vs. 7.5%, *p* = 0.002). MPN patients harbored significantly more frequent ZF2 mutations and in-frame deletions compared to other diagnostic groups (89% vs. 51%, *p* = 0.003) and (67% vs. 27%, *p* = 0.003), respectively. Thirty-two (63%) patients showed cytogenetic abnormalities by karyotyping while 19 (37%) patients were normal. The most common cytogenetic abnormalities involved chromosome 7 (3 of which were monosomy7) abnormalities (*N* = 9, 28%), trisomy 8 (*N* = 7, 22%), and 20q deletion (*N* = 6, 19%). (Supplemental Table S3).

**Fig. 1:**
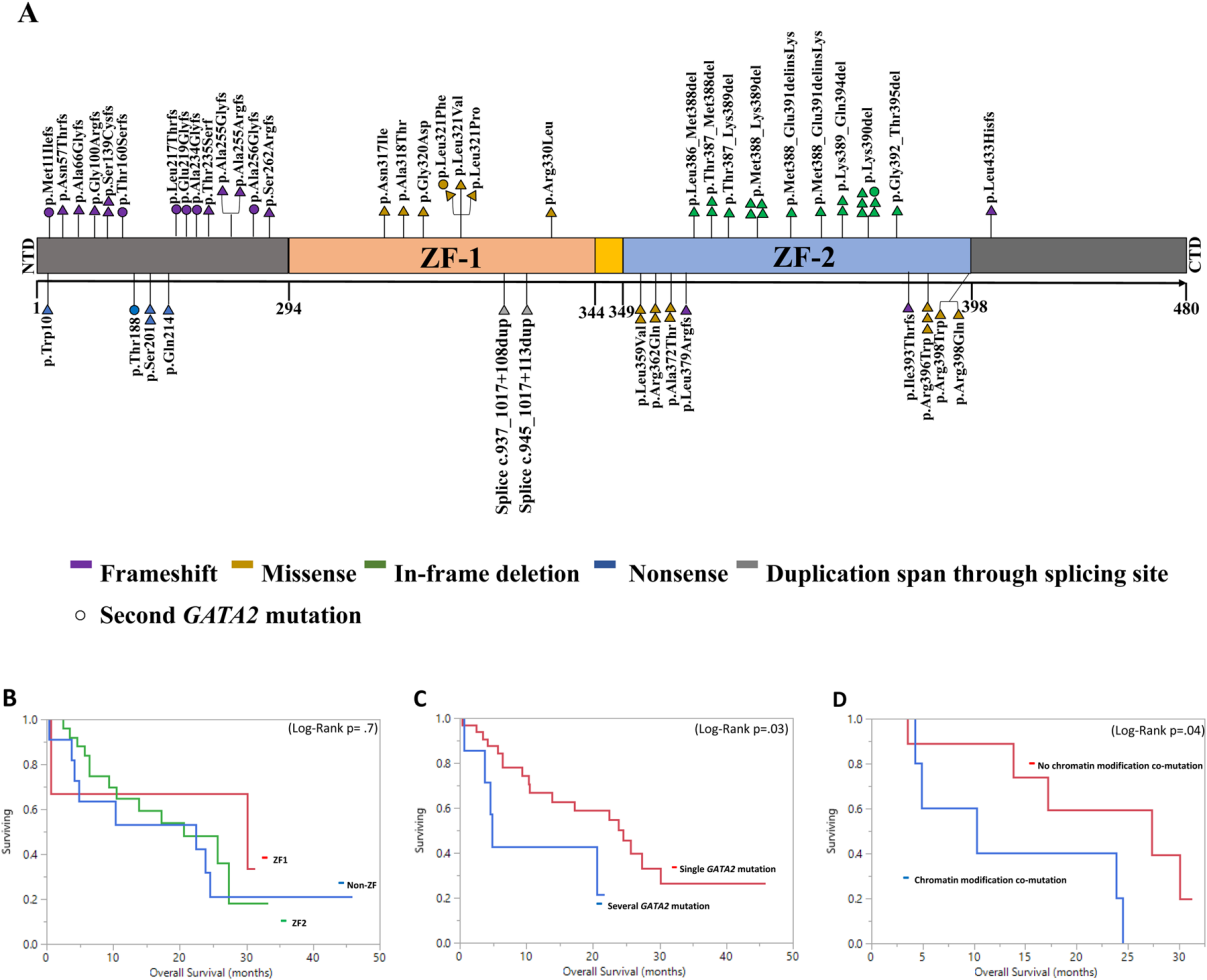
GATA2 gen mutations distribution and survival outcome in myeloid neoplasms. **A** Representation of *GATA2* variants detected, positioned on the *GATA2* protein, and its functional domains. NTD N-terminal domain, ZF zinc finger domain, CTD C-terminal domain. Kaplan–Meier survival curves for OS **B** Stratified by *GATA2* mutation location in 40 chronic myeloid neoplasm patients. There was no difference in median OS between *GATA2*-ZF1, ZF2, and non-ZF mutations (30 vs. 20.6 vs. 22.4 months, *p* = 0.7). **C** Stratified by the number of *GATA2* mutations in 40 chronic myeloid neoplasm patients. **D** Stratified by chromatin modification co-mutation status in 15 MDS patients.

We then investigated the interaction of 28 distinct co-mutations in 52 (96%) patients. *ASXL1* (60%), *SRSF2* (33%), *RUNX1* (19%), *U2AF1* (59%), *TET2* (17%), and *SF3B1* (13%) were the most common co-mutations (Supplemental Fig. S1, and Supplemental Table S4–8). Median number of mutations per patient was 4 (range = 1–6) (Supplemental Table S9). Thirty-four, 32, 24, 15, and 13 patients had at least one mutation in chromatin modification, RNA splicing, signaling, DNA methylation, and transcription pathway, respectively (Supplemental Table S10). *GATA2*-ZF2 and non-ZF had significantly higher frequency of *ASXL1* co-mutation compared to *GATA2*-ZF1 (74% vs. 11%, *p* = 0.0007) and (50% vs. 11%, *p* = 0.04), respectively. Chronic MN had significantly higher frequency of *ASXL1* co-mutation compared to AML (73% vs. 14%, *p* = 0.0001). Mean age of *GATA2-*mutated patients with *ASXL1* mutations was higher than that of patients without an *ASXL1* mutation (70 vs. 62 years, *p* = 0.02).

Two *biCEBPA* and two *KIT* mutations occurred in ZF1-mutated patients, but none were co-mutated in non ZF-1 mutated patients. *BiCEBPA*, *KIT*, and *FLT3* were co-mutated only in AML but not in chronic MN. ZF1-mutated patients had a significantly higher frequency of *SF3B1* (44% vs. 7%, *p* = 0.002) and *TET2* co-mutations (56% vs. 9%, *p* = 0.0006) than non ZF-1. ZF2-mutated patients had a significantly higher frequency of *JAK2* (19% vs. 0%, *p* = 0.02) than non ZF-2 patients.

*GATA2*-mutated MPN patients had a significantly higher frequency of *CALR*, *MPL*, and *JAK2* co-mutation (22% vs. 0%, *p* = 0.001), (11% vs. 0%, *p* = = 0.02), (33% vs. 6.7%, *p* = 0.02) than other MN. *GATA2*-mutated MDS patients had a significantly higher frequency of *BCOR* (20% vs. 3%, *p* = 0.02), and *U2AF1* (33% vs. 10%, *p* = 0.04) co-mutations, whereas MDS/MPN overlap patients showed a significantly higher frequency of *ASXL1*, *SETBP1*, and *SRSF2* co-mutation (94% vs. 42%, *p* = 0.0005), (13% vs. 0%, *p* = 0.02), (56% vs. 21%, *p* = 0.01) than other MN.

In-frame deletion *GATA2* mutation co-occurred with *ASXL1*, *SRSF2*, and *ZRSR2* mutations at a higher frequency of (89% vs. 42%, *p* = 0.0009), (50% vs. 22%, *p* = 0.03), (11% vs. 0%, *p* = 0.04) compared to other mutation types. *GATA2* missense mutation, nonsense mutation, and frameshift mutation had higher frequency of *SF3B1* (28% vs. 6%, *p* = 0.02), *EZH2* (50% vs. 6%, *p* = 0.003), and *SETBP1* (17% vs. 0%, *p* = 0.007) co-mutation compared to other mutation types, respectively.

Overall, 34 (63%) patients died after a median follow-up period of 26.4 months. The median overall survival (mOS) was 13.8 months for the overall cohort, and 23.9, 22.4, 20.6, and 5.6 months in MDS, MDS/MPN, MPN, and AML patients, respectively. There was no statistically significant difference in survival between diagnostic groups (*p* = 0.1). After excluding AML patients for OS analysis, we found that the 7 patients harboring more than one *GATA2* mutations had worse mOS (4.9 vs. 24.5 months, *p* = 0.03) (HR = 3.01, *p* = 0.04) compared to those with single *GATA2* mutation (Fig. 1). Patients with chromatin modification co-mutation had significantly worse 1-year estimated OS (1-yr-OS) (57% vs. 86%, *p* = 0.048). Multivariate analysis demonstrated that co-mutation in chromatin modification and multi-*GATA2* mutations were independent poor prognostic factors for OS with HR of 4.8 (*p* = 0.047) and HR of 3.5 (*p* = 0.02), respectively (Supplemental Table S11) in chronic MN. Similarly, MDS patients with chromatin modification co-mutation had worse mOS than their un-mutated counterparts (17 vs. 30 months, *p* = 0.04, Fig. 1). MDS patients with frameshift (FS) mutation (10.3 vs. 27.37 months, *p* = 0.048) and in-frame deletion mutation (8.7 vs. 24.5 months, *p* = 0.03) had worse mOS than non-FS mutation and non-in-frame deletion, respectively (Supplemental Fig. S2). There was no significant difference in OS in regards to mutation type in other diagnostic groups. MDS/MPN overlap patients harboring *RUNX1* co-mutation had worse mOS (2.2 vs. 25.7 months, *p* = 0.0004) compared to those without the co-mutation. Finally, although Tien et al. found that ZF1-mutated AML patients had better OS than *GATA2* ZF2-mutated patients, we could not find such difference in non-AML patients with mOS in GATA2-ZF1, ZF2, and non-ZF (30 vs. 20.6 vs. 22.4 months, *p* = 0.7), respectively (Fig. 1) [9]. Seven out of 40 (17.5 %) chronic MN patients progressed into AML with a median leukemic-free survival of 10.4 months.

Our study is limited due to the small sample size, retrospective nature, and inability to rule out the possibility of germline mutation, although the clinical and family history and/or the mutational VAF in these cases did not support a germline nature. We, for the first time, were able to investigate the interaction of different ZF domain mutations with other somatic mutations that recurrently occur in myeloid neoplasms. Our findings confirm the higher frequency of ZF1 with AML and missense mutation. In addition, they suggest that *ASXL1* mutations co-occur more frequently with chronic MN and ZF-2-*GATA2* mutations than AML and ZF-1-*GATA2* mutations, respectively. *GATA2* in-frame deletions and *JAK2* co-mutation occurred exclusively in ZF-2-*GATA2-*mutated cases, whereas non-sense mutations and duplications that span through the splicing site occurred in non-ZF and ZF-1 regions, respectively. We propose that larger cohorts are needed to validate our findings and further explore the association and implication of different somatic *GATA2* mutations.

## Supplementary information

Supplementary information
