# Supplementary material for: Clinical and biological characteristics and prognostic impact of somatic GATA2 mutations in myeloid malignancies: a single institution experience
Source: Blood Cancer J. 2021 Jun 30;11(6):122. doi: 10.1038/s41408-021-00517-0 (PMC8245641; doi:10.1038/s41408-021-00517-0)
Supplement: Supplementary file 1 — Supplementary information [file 41408_2021_517_MOESM1_ESM.pdf]

Supplemental materials and methods

This is a retrospective, single institution study within the Mayo Clinic Cancer Center. Chart review of cases with *GATA2* mutations between 5/2015 and 7/2020 was conducted after approval by the Institutional Review Board (IRB). The procedures followed were in accordance with the Declaration of Helsinki of 1975, as revised in the year 2000. In this study we included patients with pathogenic *GATA2* mutation diagnosed with a myeloid malignancy. We excluded patients with *GATA2* variants of unknown significance (VUS) without pathogenic/likely pathogenic *GATA2* mutations and patients without myeloid neoplasm. Bone marrow biopsy and aspirate were reviewed by a hematopathologist as a part of a routine clinical workflow. The diagnosis was rendered according to World Health Organization (WHO) 2016 classification of myeloid neoplasms and its revision [1, 2]. Clinical and lab results were collected from medical records. Three patients with proven germline *GATA2* mutations and two patients with *GATA* 2 VUS were excluded. In the remaining 54 patients, *GATA2* mutations were defined as likely somatic based on genetic characteristics including variant VAF and clinical history, and lack of germline genetic testing. Zinc finger 1 (ZF) corresponds to the amino acid (p.) 294-344, ZF-2 corresponds to p.349-398, and anything outside of ZF-1/ZF-2 region represents outside of the zinc finger location[3].

Mutation analysis and next generation sequencing

Next generation sequencing (NGS) panel was performed on peripheral blood or bone marrow specimens (OncoHeme, Mayo Clinic). NGS gene list up to 2018 included the following 35 genes: *ASXL1, BCOR, BRAF, CALR, CBL, CEBPA, NOTCH1, NPM1, NRAS, PHF6, PTPN11, RUNX1, SETBP1, SF3B1, TERT, TET2, TP53, U2AF1, WT1, CSF3R, DNMT3A, ETV6, EZH2, FLT3, GATA1, GATA2, IDH1, IDH2, JAK2, KIT, KRAS, MPL, MYD88*, *NOTCH1* and *ZRSR2*; in 2018, *BRAF, MYD88*, *NOTCH1* was omitted and *ANKRD26, DDX41, ELANE, ETNK1, KDM6A,*

RAD21, SH2B3, SRP72, SMC3, and STAG2 were added [4]. DNA was target-enriched with a custom hybridization-capture reagent (SureSelectXT, Agilent, Santa Clara, CA) and sequenced on the MiSeq or HiSeq platforms (Illumina, San Diego, CA) at the Mayo Clinic Clinical Genome Sequencing Laboratory. Sequencing data was processed through a custom bioinformatics pipeline Mayo NGS Workbench, using CLC Bio Genomics Server v6.0 (Qiagen, Redwood City, CA) for alignment and variant calling. The aligned BAM files were further processed through an in-house developed breakpointSearch tool for large insertion/deletion detection. BAM files of all variant calls were manually reviewed in the genome browser Alamut® Visual (Interactive Biosoftware, Rouen, France) for confirmation. The limit of detection of the NGS assay is 5% with a minimum 250X depth of coverage. More than 95% of tested regions had >1000X depth of coverage in the clinical assay. Genetic variants were curated and annotated in the Mayo Clinic Molecular Hematopathology Laboratory following the American College of Medical Genetics and Genomics (ACMG) five-tier system [5]. If a patient had more than one pathogenic/likely pathogenic mutations detected in the same gene, we included only the mutation with highest VAF value in the analysis except for the assessment of impact of the number of *GATA2* mutations.

Statistical analyses

Comparisons of categorical parameters were performed using the Chi-square test ($\chi 2$). Fisher exact test was used instead of the $\chi 2$ test when 20% of cells have expected count less than 5. For continuous variables, we used the Wilcoxon test equivalent to Mann-Whitney test. Overall survival (OS) estimates were calculated using Kaplan-Meier curves which was calculated from NGS sample collection date and the date of death due to any cause. Patients who were alive at the last follow-up date were censored at that time. Leukemia-free survival (LFS) was measured from the date of NGS until AML progression or death from any cause, whichever occurred first. P values < .05 were considered statistically significant. Multivariate Cox proportional hazard regression analysis was used to investigate independent prognostic factors for OS. All statistical analyses were performed using JMP® Pro 14.1.0 Software for Microsoft Windows.

Table S1. Characteristics and hematological features of patients with *GATA2* mutation.

**Variable**	**Value**	**Missing**
No. of patients	54	0
Age years, median(range)	67 (26 – 89)	0
Sex (male), n (%)	36 (67)	0
Hemoglobin G/DL, median (range)	8.6 (5.8 – 13)	0
Leukocytes 109/L, median (range)	12.9 (0.3 – 186)	0
Thrombocytes 109/L, median (range)	53 (1.5 – 873)	0
MCV median (range)	93 (77 – 119)	1
ANC, median (range)	6.64 (0.17 – 96.7)	1
AMC, median (range)	0.39 (0 – 44.5)	5
ALC, median (range)	1.44 (0.37 – 38.8)	2
RDW, median (range)	17.7 (13.1 – 26.8)	0
Number of mutations, median (range)	4 (1 - 6)	0
Anemia, n (%)	51 (94)	0
Leukopenia, n (%)	13 (24)	0
Thrombocytopenia, n (%)	44 (81)	0
Any cytopenia, n (%)	54 (100)	0
Pancytopenia, n (%)	12 (22)	0
Abnormal cytogenetics, n (%)	32 (63)	3

Table S2. Molecular features of 54 myeloid neoplasm patients harboring somatic *GATA2* mutations.

Case #	Nucleotide change	Amino acid change	Mutation location	Mutation type	VAF (%)	Myeloid neoplasm presentation	Co-mutation
M1	c.961C>T	p. Leu321Phe	ZF1	Missense	6	MDS	*SF3B1, TET2*
M2	c.197_200del	p.Ala66Glyfs*13	Outside ZFDs	Frameshift	19	AML	
M4	c.170del, c.1168_1170de	p.Asn57Thrfs*23, p.Lys390del	Outside ZFDs, ZF2	Frameshift, In-frame deletion	36, 33	MDS	*ASXL1, SRSF2, U2AF1*
M5	c.950A>T	p.Asn317Ile	ZF1	Missense	42	AML	*PTPN11, RUNX1, SF3B1*
M6	c.785dup	p.Ser262Argfs*20	Outside ZFDs	Frameshift	46	MDS/MPN	*ASXL1, NRAS, SETBP1, SRSF2*
M7	c.297dup	p.Gly100Argfs*85	Outside ZFDs	Frameshift	28	MDS	*BCOR*
M8	c.1174_1185del	p.Gly392_Thr395del	ZF2	In-frame deletion	37	MDS/MPN	*ASXL1, NRAS, SRSF2*
M9	c.1085G>A	p.Arg362Gln	ZF2	Missense	28	MDS/MPN	*DNMT3A, KRAS,*

						NPM1
M10 c.961C>G	p.Leu321Val	ZF1	Missense	17	AML	*SF3B1, U2AF1*
M11 c.30del	p.Trp10*	Outside ZFDs	Nonsense	30	MDS/MPN	*ASXL1, EZH2*
M12 c.1075T>G	p.Leu359Val	ZF2	Missense	12	MDS/MPN	*ASXL1, JAK2*
M13 c.1192C>T	p.Arg398Trp	ZF2	Missense	51	MDS/MPN	*ASXL1, CBL, SRSF2*
M14 c.1163_1168del	p.Met388_Lys389del	ZF2	In-frame deletion	55	MPN	*ASXL1, JAK2, RUNX1, SRSF2*
M15 c.1186C>T	p.Arg396Trp	ZF2	Missense	23	MDS	*ASXL1, U2AF1*
M16 c.1160_1165del	p.Thr387_Met388del	ZF2	In-frame deletion	36	MDS/MPN	*ASXL1, U2AF1*
M17 c.1161_1166del	p.Met388_Lys389del	ZF2	In-frame deletion	53	MDS/MPN	*ASXL1, SRSF2*
M18 c.1168_1170del	p.Lys390del	ZF2	In-frame deletion	42	MDS/MPN	*ASXL1, KRAS,*

							SRSF2
M19	c.416_417del	p.Ser139Cysfs*45	Outside ZFDs	Frameshift	7	AML	*DNMT3A, TP53*
M21	c.1156_1164del	p.Leu386_Met388del	ZF2	In-frame deletion	44	MDS	*ASXL1, ZRSR2*
M22	c.1075T>G	p.Leu359Val	ZF2	Missense	42	MDS	*ASXL1, BCOR, U2AF1*
M23	c.1085G>A	p.Arg362Gln	ZF2	Missense	44	AML	*DNMT3A, ETV6, FLT3, RUNX1*
M24	c.1163_1168del	p.Met388_Lys389del	ZF2	In-frame deletion	28	MPN	*ASXL1, CALR*
M25	c.962T>C	p.Leu321Pro	ZF1	Missense	44	AML	*CEBPA, KIT, NRAS, WT1*
M26	c.1168_1170del, c.766dup	p.Lys390del, p.Ala256Glyfs*26	ZF2, Outside ZFDs	In-frame deletion, Frameshift	74, 6	MPN	*ASXL1, SRSF2*
M28	c.1186C>T	p.Arg396Trp	ZF2	Missense	31	MDS	*ASXL1, RUNX1*

M29	c.1160_1168del	p.Thr387_Lys389del	ZF2	In-frame deletion	44	AML	*ASXL1, ETV6, NRAS, SRSF2*
M30	c.989G>T, c.961C>T	p.Arg330Leu, p.Leu321Phe	ZF1	Missense	17, 7	MDS/MPN	*ASXL1, EZH2, KRAS, RUNX1, TET2*
M31	c.1296_1306del	p.Leu433Hisfs*99	Outside ZFDs	Frameshift	5	MPN	*ASXL1, CALR, TET2*
M32	c.1160_1165del, c.701_708de	p.Thr387_Met388del, p.Ala234Glyfs*45	ZF2, Outside ZFDs	In-frame deletion, Frameshift	33.8, 32	MPN	*ASXL1, SF3B1, SRSF2*
M33	c.1163_1171del	p.Met388_Glu391delinsLys	ZF2	In-frame deletion	46	AML	*JAK2, KRAS, RUNX1*
M34	c.1168_1170del, c.32dup	p.Lys390del, p.Met11Ilefs*174	ZF2, Outside ZFDs	In-frame deletion, Frameshift	68, 9	AML	*ASXL1, FLT3, SRSF2, TET2*
M35	c.700_703dup	p.Thr235Serfs*48	Outside ZFDs	Frameshift	31	MDS	*BCOR, RUNX1,*

							U2AF1
M36	c.640C>T	p.Gln214*	Outside ZFDs	Nonsense	41	MDS/MPN	*ASXL1, EZH2, TET2*
M37	c.1114G>A	p.Ala372Thr	ZF2	Missense	5	MPN	*BCOR, EZH2, JAK2, SF3B1, TP53*
M38	c.599dup	p.Ser201*	Outside ZFDs	Nonsense	39	AML	*KRAS, NRAS*
M39	c.1161_1166del	p.Met388_Lys389del	ZF2	In-frame deletion	15	MDS/MPN	*ASXL1, JAK2, KDM6A, U2AF1*
M40	c.1176_1188del	p.Ile393Thrfs*80	ZF2	Frameshift	73	MDS/MPN	*ASXL1, SETBP1, SRSF2*
M42	c.1136del, c.648dup	p.Leu379Argfs*8, p.Leu217Thrfs*65	ZF2, Outside ZFDs	Frameshift, Frameshift	31, 8	MDS/MPN	*ASXL1, SRSF2, STAG2*
M43	c.1165_1170del	p.Lys389_Lys390del	ZF2	In-frame deletion	48	MPN	*ASXL1, ZRSR2*

M46	c.599dup	p.Ser201*	Outside ZFDs	Nonsense	31	MDS	*NPM1*
M47	c.945_1017+113 dup	p.?	ZF1	Duplication span through splicing site	11	MDS	*SRSF2, TET2*
M48	c.959G>A	p.Gly320Asp	ZF1	Missense	26	AML	*CEBPA, TET2*
M49	c.1168_1170del AAG	p.Lys390del	ZF2	In-frame deletion	41	MPN	*ASXL1, MPL*
M50	c.952G>A	p.Ala318Thr	ZF1	Missense	15	AML	*SF3B1, STAG2*
M51	c.1186C>T	p.Arg396Trp	ZF2	Missense	8	MPN	*DNMT3A, JAK2, STAG2, U2AF1*
M52	c.1168_1170del, c.654_655dup	p.Lys390del, p.Glu219Glyfs*4	ZF2, Outside ZFDs	In-frame deletion, Frameshift	39, 34	MDS/MPN	*ASXL1, SRSF2*
M53	c.763dup	p.Ala255Glyfs*27	Outside ZFDs	Frameshift	24	MDS	*WT1*
M54	c.1166_1183del	p.Lys389_Gln394del	ZF2	In-frame deletion	26	MDS	*ASXL1, NRAS, U2AF1*
M55	c.937_1017+108 dup		ZF1	Duplication span through splicing site		AML	*KIT, RUNX1, SRSF2,*

							TET2
M56	c.1114G>A	p.Ala372Thr	ZF2	Missense	32	AML	
M57	c.1166_1183del	p.Lys389_Gln394del	ZF2	In-frame deletion	23	MDS	*SF3B1*
M58	c.1193G>A	p.Arg398Gln	ZF2	Missense	47	MDS	*ETV6, RUNX1*
M59	c.416_417del	p.Ser139Cysfs*45	Outside ZFDs	Frameshift	18	MDS	*ASXL1, CSF3R, EZH2, TET2*
M61	c.761_762dup, c.555_561 dup, c. 476_477dup	p. Ala255Argfs*72, p.Thr188*, p.Thr160Serfs*59	Outside ZFDs	Frameshift	13, 8, 12	MDS/MPN	*ASXL1, RUNX1,* SRSF2

Abbreviations: MDS, myelodysplasia neoplasm; AML, acute myeloid leukemia; MPN, Myeloproliferative neoplasms; MDS/MPN, Myelodysplastic/Myeloproliferative neoplasms; NGS, next generation sequencing; ZF, zing finger; ZFD, zing finger domain; VAF, *GATA2* variant allele frequencies.

Table S3. Cytogenetic abnormalities in patients with *GATA2* mutations.

Patient Number	Cytogenetic abnormality	Myeloid neoplasm type
M1	46, XX, -2, -20, +2mar [2]/46, XX [18]	MDS
M 2	46, XX, t (9;11) (p22; q23) [19]/46, XX [1]	AML
M 4	45, XY, -7[6]/46, XY, del (7) (q11.2) [1]/46, XY [13]	MDS
M 5	47, XY, +12[3]/47, sl, +1, der (1;20) (q10; p10) [1]/46, XY [16]	AML
M 6	47, XX, +8[4]/46, XX [16]	MDS/MPN
M 7	47, XY, +8[14]/46, XY [6]	MDS
M 8	46, XX, +14, der (14;18) (q10; q10) [20]	MDS/MPN
M 13	46, XY, i (17) (q10) [5]/47, idem, +13[15]	MDS/MPN
M 14	46, XY, del (7) (q22q34) [4]/46, XY [16]	MPN
M 19	43-46,XY,del(4)(q25),del(5)(q31q35), -7,add(8)(q22),del(12)(q24.1),dic(15;20)(p11.2;q13.1),add(17)(p11.2),psu dic(18;6)(q23;q13),+1-2r,+mar[cp16]/52 60, XY,+Y,+1,+2,del(5)(q31q35),+6,+add(8)(q22),+10,add(12)(p11.2),-15, add(15)(p11.2),+18,+hsr(19)(p13.1),+21,+21,add(22)(q11.2),+1-2r,+1-4mar[cp4]	AML
M 21	46,XY,del(7)(q22)[7]/47,sl,+13[2]/47,sl,+21[1]/47,XY,+21 [2]/48,sl2,+13[4]/47,XY,del(7)(q22q34),+13[3]/46,XY[1].	MDS
M 22	46, XY, del (5) (q13q33) [9]/46, XY [11]	MDS
M 23	46, XX, t (2;14) (q21; q32) [18]/46, XX [2]	AML
M 28	46, XX, +1, der (1;22) (q10; q10), del (20) (q11.2q13.1) [20]	MDS
M 29	47, XY, +13[2]/46, XY [18].	AML
M 33	46, XY, add (11) (p11.2) [20]	AML
M 34	46, XY, del (3) (q13.2q25) [15]/46, XY [5]	AML
M 35	46, XX, der (7)t(1;7) (q21; q34) [4]/47, idem, +8[10]/46, XX [1]	MDS
M 36	50, XX, -8, +9, +11, +14, +19, +22[1]/46, XX [19].	MDS/MPN
M 37	46, XY, del (13) (q12q22) [1]/46, XY [19]	MPN
M 38	t (11;19) in 20/20 metaphases	AML
M 39	46, XY, del (7) (q22q34) [20].	MDS/MPN
M 42	46, XY, del (20) (q11.2q13.3) [1]/46, XY [19]	MDS/MPN
M 47	46, XX, -7, +r [7]/46, XX [13]	MDS
M 49	46, XY, del (20) (q11.2q13.3) [20].	MPN
M 51	46, XY, t (3;12) (q26.2; p13), del (20) (q11.2q13.3.) [10]	MPN
M 53	47, XY, t (11;19) (q23; p13.1), +15[15]//46, XX [5]	MDS

M 54	46, XY, add (7) (q11.2) [20]	MDS
M 56	46, XY, t (9;10) (q34; q22) [20]	AML
M 57	46, XY, +1, der (1;7) (q10; p10) [6]/47, idem, +8[14]	MDS
M 58	47, XX, +8[19]/46, XX [1]	MDS
M 61	47, XY, +8[10]/46, XY [10]	MDS/MPN

Abbreviations: MDS, myelodysplasia neoplasm; AML, acute myeloid leukemia; MPN, Myeloproliferative neoplasms; MDS/MPN, Myelodysplastic/Myeloproliferative neoplasms.

Table S4. Mutation frequency and variant allele frequencies (VAF) distribution in patients with *GATA2* mutation.

**Gene**	**N**	**Median**	**Minimal, %**	**Maximum, %**
GATA2	54*	31%	5%	74%
ASXL1	31	40%	12%	57%
SRSF2	17*	42%	18%	51%
RUNX1	10*	37%	15%	49%
U2AF1	9	42%	18%	47%
TET2	9*	38%	6%	56%
SF3B1	7	28%	10%	48%
JAK2	6	68%	34%	100%
NRAS	6	28%	9%	36%
EZH2	5	44%	41%	93%
KRAS	5	31%	22%	47%
BCOR	4	29%	8%	65%
ETV6	3	25%	16%	45%
DNMT3A	4	45%	35%	45%
SETBP1	2	47%	45%	48%
STAG2	3	28%	14%	50%
CEBPA	2	40%	35%	45%
TP53	2	18%	7%	28%
ZRSR2	2	77%	61%	92%
WT1	2	28%	19%	37%
FLT3	2	47%	37%	57%
CALR	2	52%	49%	54%
NPM1	2	45%	42%	48%
CBL	1	46%		
PTPN11	1	42%		
KDM6A	1	8%		
MPL	1	24%		
KIT	2*	12%		
CSF3R	1	23%		

*1 missing VAF value

Table S5. Mutation frequency and variant allele frequencies (VAF) distribution in acute myeloid leukemia patients with *GATA2* mutations.

Gene	N	Median%	Minimal, %	Maximum, %
GATA2	14*	39%	7%	68%
RUNX1	4*	43%	38%	49%
SRSF2	3*	47%	47%	47%
TET2	3*	40%	35%	44%
SF3B1	3	20%	18%	45%
NRAS	3	22%	9%	35%
ASXL1	2	50%	42%	57%
KRAS	2	32%	22%	41%
ETV6	2	35%	25%	45%
DNMT3A	2	40%	35%	45%
CEBPA	2	40%	35%	45%
FLT3	2	47%	37%	57%
KIT	2*	12%		
U2AF1	1	18%		
JAK2	1	100%		
STAG2	1	28%		
TP53	1	28%		
WT1	1	37%		
PTPN11	1	42%		
EZH2	0			
BCOR	0			
SETBP1	0			
ZRSR2	0			
CALR	0			
NPM1	0			
CBL	0			
KDM6A	0			
MPL	0			
CSF3R	0			

*1 missing VAF value

Table S6. Mutation frequency and variant allele frequencies (VAF) distribution in myelodysplastic syndromes patients with *GATA2* mutation.

Gene	N	Median%	Minimal, %	Maximum, %
GATA2	15	28%	6%	49%
ASXL1	7	37%	36%	45%
U2AF1	5	41%	24%	45%
RUNX1	3	30%	15%	32%
TET2	3	13%	10%	56%
BCOR	3	35%	8%	65%
SRSF2	2	25%	18%	32%
SF3B1	2	19%	10%	28%
ETV6	1	16%		
NRAS	1	15%		
EZH2	1	93%		
ZRSR2	1	92%		
WT1	1	19%		
NPM1	1	48%		
CSF3R	1	23%		
SETBP1	0			
JAK2	0			
KRAS	0			
DNMT3A	0			
STAG2	0			
CEBPA	0			
TP53	0			
FLT3	0			
CALR	0			
CBL	0			
PTPN11	0			
KDM6A	0			
MPL	0			
KIT	0			

Table S7. Mutation frequency and variant allele frequencies (VAF) distribution in myelodysplastic/myeloproliferative neoplasms patients with *GATA2* mutation.

Gene	N	Median, %	Minimal, %	Maximum, %
GATA2	16	37%	12%	73%
ASXL1	15	40%	12%	56%
SRSF2	9	45%	38%	51%
EZH2	3	43%	41%	45%
KRAS	3	31%	25%	47%
RUNX1	2	40%	37%	43%
U2AF1	2	45%	42%	47%
TET2	2	27%	6%	47%
JAK2	2	68%	63%	72%
NRAS	2	35%	34%	36%
SETBP1	2	47%	45%	48%
DNMT3A	1	45%		
STAG2	1	14%		
NPM1	1	42%		
CBL	1	46%		
KDM6A	1	8%		
SF3B1	0			
BCOR	0			
ETV6	0			
CEBPA	0			
TP53	0			
ZRSR2	0			
WT1	0			
FLT3	0			
CALR	0			
PTPN11	0			
MPL	0			
KIT	0			
CSF3R	0			

Table S8. Mutation frequency and variant allele frequencies (VAF) distribution in myeloproliferative neoplasms patients with *GATA2* mutation.

Gene	N	Median, %	Minimal, %	Maximum, %
GATA2	9	34%	5%	74%
ASXL1	7	41%	17%	52%
SRSF2	3	47%	46%	49%
JAK2	3	41%	34%	100%
SF3B1	2	42%	36%	48%
CALR	2	52%	49%	54%
RUNX1	1	29%		
U2AF1	1	42%		
TET2	1	41%		
EZH2	1	44%		
BCOR	1	23%		
DNMT3A	1	44%		
STAG2	1	50%		
TP53	1	7%		
ZRSR2	1	61%		
MPL	1	24%		
NRAS	0			
KRAS	0			
ETV6	0			
SETBP1	0			
CEBPA	0			
WT1	0			
FLT3	0			
NPM1	0			
CBL	0			
PTPN11	0			
KDM6A	0			
KIT	0			
CSF3R	0			

Table S9. Number of total mutations per patient.

Number of total mutations per patient	Count (N)
1	2
2	4
3	20
4	16
5	10
6	2

Table S10. Pathway pattern of co-mutations in 52 *GATA2*-mutated patients.

**Type**	**Co-mutations in set, N (%)**
DNA methylation<br>*DNMT3A, TET2, IDH2, IDH1, WT1*	15 (29)
RNA splicing<br>*ZRSR2, U2AF1, SRSF2, SF3B1*	32 (62)
Transcription<br>*RUNX1, ETV6, CEBPA, CEBPA*	13 (25)
Chromatin modification<br>*EZH2, ASXL1, KDM6A, BCOR*	34 (65)
signaling<br>*NRAS, CBL, BRAF, CALR, MPL, KIT, PTPN11, JAK2, CSF3R, KRAS, FLT3*	24 (46)

Table S11. Multivariate analysis (Cox regression) on the OS in 40 *GATA2-mutated chronic myeloid neoplasm* patients

Variables	RR	95% CI	P value
Age>67	2.8	1.14-6.8	0.02
Chromatin modification pathway mutation	4.8	1.01-22.6	0.047
Several *GATA2* mutation	3.5	1.1-10.9	0.02

Abbreviations: RR, risk ratio; CI, confidence interval; VAF, Variant Allele Frequencies.

Figure S1. Frequency of co-mutations (n) identified in 54 *GATA2*-mutated patients.

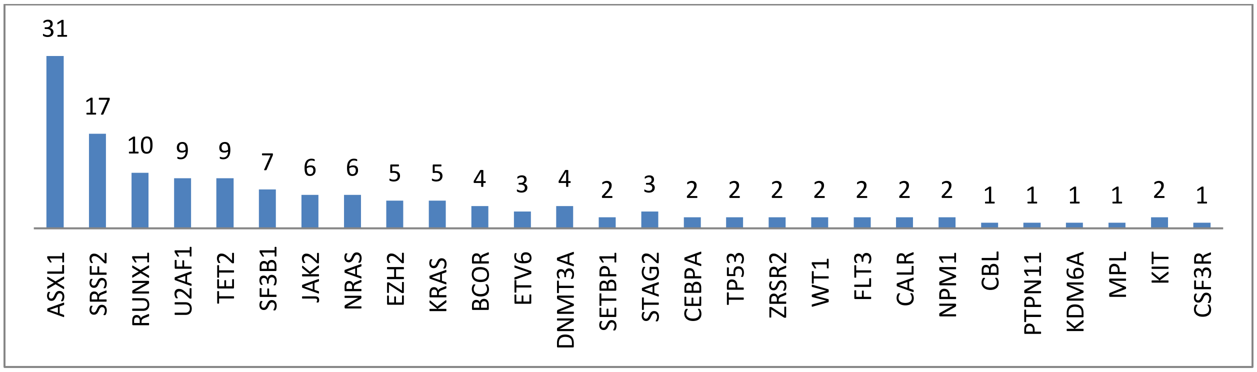

Figure S2. Kaplan–Meier survival curves for OS (A) Stratified by frameshift mutation status in 15 MDS patients. (B) Stratified by in-frame deletion mutation status in 15 MDS patients.

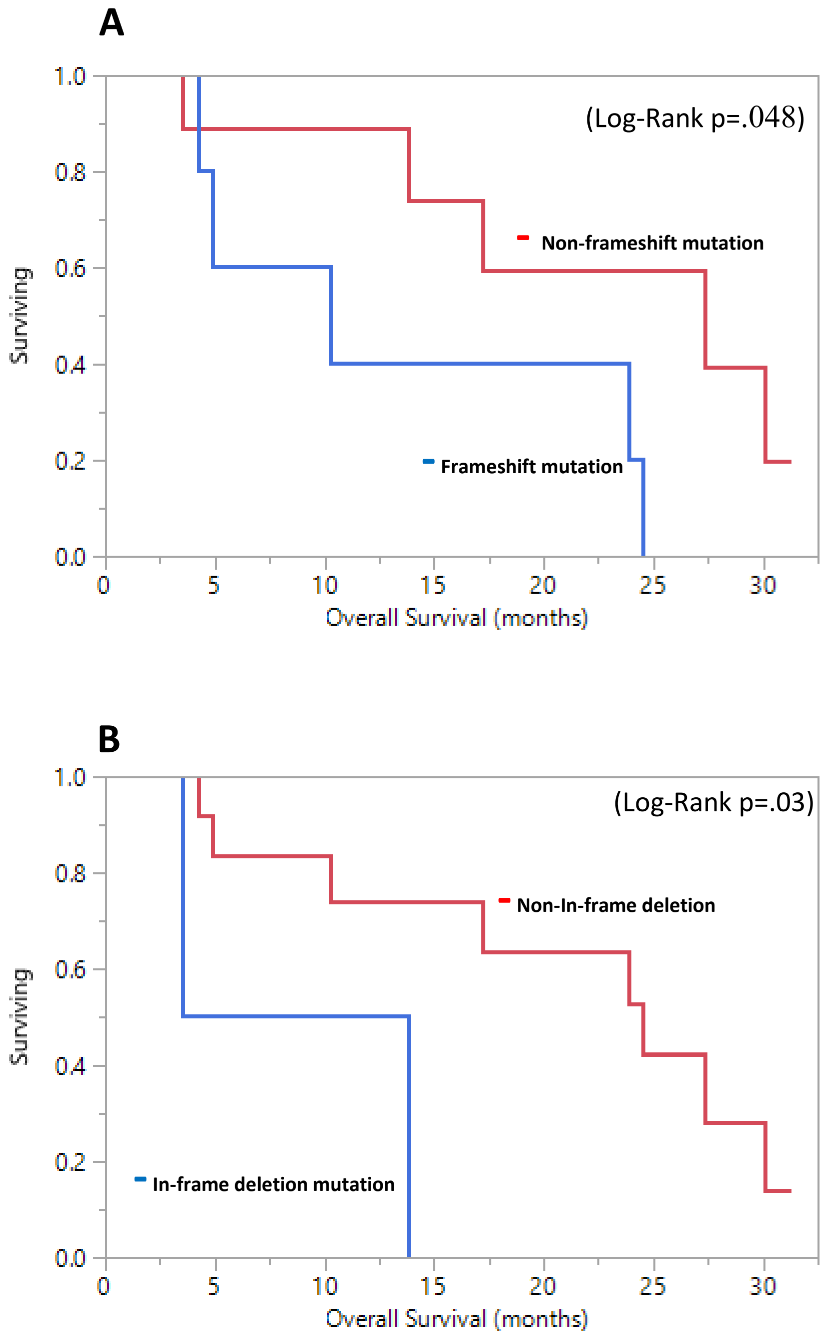

Refrences

1. Arber DA. et al. The 2016 revision to the World Health Organization classification of myeloid neoplasms and acute leukemia. Blood. 2016;127(20):2391-405.
2. Vardiman JW, Harris NL, Brunning RD. The World Health Organization (WHO) classification of the myeloid neoplasms. Blood. 2002;100(7):2292-302.
3. Hyde RK, Liu PP. GATA2 mutations lead to MDS and AML. Nature Genetics. 2011;43(10):926-7.
4. He R. et al. Hybridization capture-based next generation sequencing reliably detects FLT3 mutations and classifies FLT3-internal tandem duplication allelic ratio in acute myeloid leukemia: a comparative study to standard fragment analysis. Modern Pathology. 2020;33(3):334-43.
5. Richards S. et al. Standards and guidelines for the interpretation of sequence variants: a joint consensus recommendation of the American College of Medical Genetics and Genomics and the Association for Molecular Pathology. Genetics in Medicine. 2015;17(5):405-23.
